# Impact of residual coronary lesions on outcomes of myocardial infarction patients with multi-vessel disease

**DOI:** 10.1186/s12872-023-03657-2

**Published:** 2024-01-23

**Authors:** Tarek A. N. Ahmed, Amr A. A. Othman, Salwa R. Demitry, Khaled M. Elmaghraby

**Affiliations:** Department of Cardiovascular Medicine, Assiut University Heart Hospital, Assiut University, Assiut, 71526 Egypt

**Keywords:** Residual SYNTAX Score (rSS), SYNTAX Revescularization Index (SRI), Acute Myocardial Infarction, Coronary Artery Disease, Residual Coronary Artery Disease

## Abstract

**Background:**

The residual burden of coronary artery disease (CAD) after percutaneous coronary intervention (PCI) drew a growing interest. The residual SYNTAX Score (rSS) was a strong prognostic factor of adverse events and all-cause mortality in patients who underwent PCI. In addition, the SYNTAX Revascularization Index (SRI), a derivative of rSS, was used to figure out the treated proportion of CAD and could be used as a prognostic utility in PCI for patients with multi-vessel disease (MVD).

**Purpose:**

We aimed at the assessment of the use of rSS and the SRI as predictors of in-hospital outcomes and up to two-year cumulative follow-up outcomes in patients with MVD who had PCI for the treatment of ST-Elevation Myocardial Infarction (STEMI) or Non-STEMI (NSTEMI).

**Methods:**

We recruited 149 patients who had either STEMI or NSTEMI while having MVD and received treatment with PCI. We divided them into tertiles based on their rSS and SRI values. We calculated baseline SYNTAX Score (bSS) and rSS using the latest version of the calculator on the internet, and we used both scores to calculate SRI. The study end-points were In-hospital composite Major Adverse Cardiovascular Events (MACE) and its components, in-hospital death, and follow-up cumulative MACE up to 2 years.

**Results:**

Neither rSS nor SRI were significant predictors of in-hospital adverse events, while female sex, hypertension, and left ventricular ejection fraction were independent predictors of in-hospital MACE. At the two-year follow-up, Kaplan-Meyer analysis showed a significantly increased incidence of MACE within the third rSS tertile (rSS > 12) compared to other tertiles (log rank *p* = 0.03). At the same time, there was no significant difference between the three SRI tertiles. Unlike SRI, rSS was a significant predictor of cumulative MACE on univariate Cox regression (HR = 1.037, *p* < 0.001). On multivariate Cox regression, rSS was a significant independent predictor of two-year cumulative MACE (HR = 1.038, *p* = 0.0025) along with female sex, hypertension, and left ventricular ejection fraction. We also noted that all patients with complete revascularization survived well throughout the entire follow-up period.

**Conclusions:**

Neither rSS nor SRI could be good predictors of in-hospital MACE, while the rSS was a good predictor of MACE at two-year follow-up. Patients with rSS values > 12 had a significantly higher incidence of cumulative MACE after 2 years. The best prognosis was achieved with complete revascularization.

## Introduction

About 40-70% of patients with ST-elevation Myocardial Infarction (STEMI) who underwent primary percutaneous coronary intervention (PPCI) were found to have significant non-culprit coronary artery stenotic lesions [1, 2]. It is known that patients with multi-vessel disease (MVD) demonstrated much worse clinical outcomes, and the risk increased even further on the occurrence of acute STEMI in terms of recurrent ischemic events and mortality [3, 4]. Nevertheless, the prognostic impact of MVD on STEMI could be quite variable because of differences in coronary artery disease (CAD) characteristics in different patients [4, 5].

The current guideline recommendations state that only the infarct-related artery should be revascularized [6]. However, there are many randomized-controlled trials (RCTs) that suggested a strategy of complete revascularization, either during the primary PCI procedure or in a staged manner, which might be beneficial and safe in a selected population of STEMI patients [7–10]. The recommendation class regarding multi-vessel PCI in hemodynamically stable patients with STEMI has been changed and upgraded from Class III (Not recommended) to class IIa (Should be considered) to include a consideration of a planned, staged multi-vessel PCI procedure before hospital discharge [11].

High-risk patients with non-ST-elevation myocardial infarction (NSTEMI) have a better prognosis if they receive early invasive treatment regarding cardiovascular mortality and re-infarction. About 50% of these patients have multi-vessel coronary artery disease, and the latest guideline recommendations demonstrated a preference for complete revascularization in this patient population [12, 13].

A growing interest in residual disease burden was observed after performing culprit-vessel/lesion PCI. The residual SYNTAX score, described in detail by Généreux and colleagues [14], was a strong prognostic factor of coronary events and all-cause death in patients who have undergone PCI. Other groups validated this score afterward and demonstrated its good prognostic accuracy for adverse ischemic events after performing PCI [15].

The SYNTAX Revascularization Index (SRI), which takes into account the severity and extent of baseline CAD (as assessed by the baseline SYNTAX score [bSS]) and the residual CAD after PCI (as assessed by the rSS), has been used in determining the proportion of CAD that has been treated and has been shown to have prognostic utility in PCI for MVD [16].

## Aim of the study

To evaluate the use of the rSS and the SRI as predictors of in-hospital (primary end-point) and long-term (secondary end-points) major adverse cardiac events (MACE) among patients with multi-vessel disease (MVD) who underwent PCI in the setting of STEMI or NSTEMI.

## Patients and methods

### Study design and population

This is a single-center, prospective observational cohort study conducted at Assiut University Heart Hospital (AUHH).

#### Patient population

All adult patients admitted to Assiut University Heart hospitals diagnosed with acute coronary syndrome (ACS), including patients with acute ST-segment Elevation Myocardial Infarction (STEMI) or Non-ST-segment Myocardial Infarction (NSTEMI) who underwent percutaneous coronary intervention (PCI) in the setting of multi-vessel disease (MVD), which was defined as significant coronary artery stenosis in two or more segments of the coronary artery tree, in the period between July 1, 2018, to December 31, 2019.

Patients were excluded if they received fibrinolytic therapy, presented with cardiogenic shock, underwent CABG, or had severe renal impairment.

**Fig. 1:**
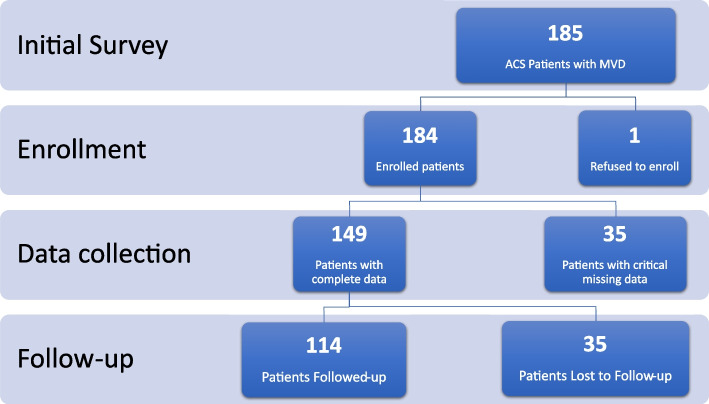
Patient study flow diagram

Within the previously mentioned criteria, 185 patients were surveyed, of which 149 patients were included, one refused to participate in the study, and 35 patients had crucial missing data. Of the 149 enrolled patients, 114 of them (76.5%) were either successfully followed up or met the primary end-point of the study, and 35 patients (23.5% of the total enrolled patients) were lost to follow-up due to the constraints imposed by the COVID-19 pandemic (Fig. 1).

#### Sample size estimation

Considering the study’s primary end-point (In-Hospital MACE rate of 28%) [17], the estimated sample size of the population, based on the power of 85% and alpha = 5%, was calculated to be 108 patients. The sample size was calculated using the OpenEpi sample size calculator: https://www.openepi.com/SampleSize/SSCohort.htm.

### Diagnosis of ACS

Patients were diagnosed with ACS based on the latest guidelines for diagnosing STEMI and NSTE-ACS published by the European Society of Cardiology (ESC) in the years 2015 and 2020 for NSTE-ACS diagnosis [13, 18] and in 2017 for STEMI diagnosis [11].

### Angiographic analysis

Angiographic analysis was performed in two orthogonal views according to the local protocols used in Assiut University Heart Hospital. The patients’ angiographic views were reviewed, and the bSS and rSS were calculated using a web-based calculator (www.syntaxscore2020.com). All the parameters and stenoses were assessed visually. The rSS was determined as the SS remaining after the completion of PCI. In the case of staged PCI procedures (defined as a second planned PCI procedure after the initial intervention), the final planned procedure was used as the entry point for this study.

### Procedural data

Patients who participated in the study with STEMI underwent PCI within 24 hours of symptoms onset, and patients with NSTEMI underwent early invasive PCI strategy, both according to the ESC guidelines, as mentioned before [11, 13, 18]. All patients who underwent PCI received 300 mg of aspirin and a 600 mg loading dose of clopidogrel or 180 mg of ticagrelor. Heparin was administered throughout the procedure according to standardized protocols. Per the operator’s decision, PCI was performed via a femoral or radial approach. Glycoprotein IIb/IIIa inhibitors were used at the discretion of the operator. After the procedure, all patients received 75-100 mg/day of aspirin indefinitely, as well as 150 mg for 2 weeks and then 75 mg/day of clopidogrel or 90 mg b.i.d. of ticagrelor for at least 12 months. Standard post-intervention care was implemented. All patients consented to the procedure.

#### Angiographic scores

##### Residual SYNTAX score

The baseline SYNTAX (bSS) score and the residual SYNTAX score (rSS) were calculated by summing up the individual scores for each lesion with diameter stenosis ≥50% in vessels with a diameter ≥ 1.5 mm in the angiography obtained before and after the procedure. The SYNTAX algorithm of scoring is fully described elsewhere [19].

##### SYNTAX revascularization index

The SYNTAX Revascularization Index (SRI), representing the proportion of CAD burden treated by PCI, was calculated using the following formula: SRI = (1-[rSS/bSS]) × 100 [20].

The bSS and rSS were calculated using both the original SYNTAX Score Calculator, currently found on http://syntaxscore.org, and the web version of the updated SYNTAX Score 2020 Calculator, which is found on http://syntaxscore2020.com. The results from both calculators were used for test-retest reliability analysis, while the results from the updated edition were used for the remainder of the data analysis. In addition, inter-rater reliability was assessed with the help of two of our colleagues at the Department of Cardiology, who calculated baseline and residual SYNTAX I scores for 10 randomly selected patients.

#### Patients’ follow-up and data collection

Patient data, including contact information, PCI reports, discharge reports, and other relevant data sources, were obtained from the AUHH database. Coronary angiography imaging data and loops were obtained from the AUHH coronary catheterization lab imaging database and Assiut University Hospitals’ Paxera® Picture Archiving and Communication Servers (PACS). Patients with missing reports or imaging data were excluded from the analysis. Patient follow-up has been performed by outpatient clinic visits. Patients lost to follow-up were excluded from the follow-up analysis, including survival analysis.

Major Adverse Cardiac Events (MACE), including in-hospital and up to 2-year follow-up MACE, represented a composite of cardiac death (including periprocedural), non-fatal myocardial infarction, heart failure (HF), unplanned revascularization including target vessel revascularization (TVR), target lesion revascularization (TLR) and Coronary artery bypass graft (CABG).

For surviving patients, the initial time limit for follow-up was 1 year after the date of the coronary intervention. However, due to logistic delays in the study, an additional 12-month follow-up was introduced to benefit from the added time for qualified patients.

#### End-points of the study and their definitions

##### Primary end-point

The primary end-point of the study was the in-hospital major adverse cardiac events (MACE).

##### Secondary end-point

Included the individual components of the primary end-point, as well as ACUITY-defined major bleeding [21] and acute kidney injury (AKI). Also, the secondary end-point included up to 2-year MACE and its individual components.

An event was characterized as periprocedural death if it occurred within 24 hours of PPCI. Pre-specified definitions of recurrent MI and bleeding were the same as those used in the ACUITY trial [21]. HF was defined as symptoms of dyspnea attributed to pulmonary congestion resulting in administering oxygen and/or intravenous diuretics, amongst other ant-failure treatments. AKI was described as a 25% relative or 0.5 mg/dL (44.2 μmol/L) absolute increase in presenting serum creatinine after PPCI [22].

In the case of staged PCI procedures (defined as a second planned PCI procedure after the initial intervention) [23], the final planned procedure was used as the entry point for this study. We used the first planned procedure as the reference if there was any event between the procedures. The rSS and SRI were calculated after all staged/planned PCI procedures were completed unless an event occurred between the procedures; then, we used the initial scores.

### Statistical analysis

All statistical analyses were performed using IBM® Statistical Package for Social Sciences (SPSS)® Statistics version 25 (SPSS Inc., Chicago, IL, USA) and MedCalc® Statistical Software version 20.013 (MedCalc Software Ltd., Ostend, Belgium; https://www.medcalc.org; 2021). Categorical data were presented as frequencies and percentages, and Chi-square tests were used to compare groups. Continuous data were reported as means ± standard deviations and medians (interquartile range), which were tested for normality using the Shapiro-Wilk test. Where continuous data were normally distributed, the Student’s T-test and One-way ANOVA were used for comparisons between groups; where data were non-normally distributed, the Mann-Whitney U and Kruskal-Wallis H tests were used. Regression analyses were performed using the binary logistic regression test. The diagnostic accuracy of the angiographic and clinical scores was assessed with the area under the curve (AUC) analysis of receiver operating characteristic (ROC) curves. Regression models were further evaluated using the concordance index (C-index) for their discrimination power. Patients were stratified into three tertiles according to their rSS and SRI values based on the percentiles of both scores.

For survival analysis, the outcome related to time from admission until death/end-point along the follow-up period was tested. The Kaplan–Meier survival curves were performed, differences in MACE rates were analyzed by log-rank test, and pairwise comparisons were performed using the Mantel-Cox test. Univariate Cox regression analyses were conducted to detect MACE’s hazard ratio (HR), and potentially relevant factors were included in a multivariate Cox regression model and adjusted for confounding patient factors. In all statistical tests, *p*-value < 0.05 was considered statistically significant.

## Study results

### Patients’ demographics and basal characteristics

The study included 149 patients who were enrolled between July 1, 2018, and December 31, 2019. The mean age for the study participants was 60.12 (± 11.77) years. Patients with STEMI who underwent PCI had a pain onset to presentation time of 6.69 (± 5.64) hours.

Table 1 shows the overall characteristics of the study sample. Within the study sample, 47 (32.5%) were females. Regarding the known cardiovascular risk factors, 67 (45%) of the patients were hypertensive, 52 (34.9%) were diabetic, 67 (45%) had a history of present or previous smoking, and 83 (55.7%) had a history of dyslipidemia.

**Table 1:** Characteristics of the overall study sample

Variable	Overall Study Sample (*n* = 149)
**Age**	60.12 (±11.77) 60 (53 – 66.5)
**Sex**	**Male:** 102 (68.5%) **Female:** 47 (32.5%)
**BMI**	27.81 (±4.45) 27.5 (24.45 – 29.4)
**Dyslipidemia**	83 (55.7%)
**Smoking**	67 (45%)
**Hypertension**	67 (45%)
**Diabetes**	52 (34.9%)
**Pain onset to presentation time in hours (Within STEMI patients) (*n* = 117)**	6.69 (±5.64) 6 (3 – 8.25)
**STEMI/NSTEMI**	117 (78.5%)**/**32 (21.5%)
**KILLIP Classification**	**Class I:** 125 (83.9%) **Class II:** 15 (10.1%) **Class III:** 2 (1.3%) **Class IV:** 7 (4.7%)
**ST Resolution (Within STEMI Patients) (*n* = 117)**	**< 30%:** 10 (8.5%) **30-70%:** 70 (59.8%) **> 70%:** 30 (25.6%)
**Significant ST Resolution (within STEMI patients) (*n* = 117)**	**100 (85.5%)**
**Echocardiography**	
**Ejection Fraction**	51.9% (± 10.88)
**Myocardial Wall Motion Score Index**	1.42 (± 0.34)
**GRACE Scores**	
**In-Hospital GRACE Score**	149.62 (± 29.9)
**Post-Discharge GRACE Score**	116.78 (± 25.15)
**Laboratory Data**	
**Total CK on Admission**	559.5 (233.75 – 1816.5)
**Peak CK value**	1162.5 (363.25 – 2446.75)
**CK-MB on Admission**	92 (40 – 232)
**Peak CK-MB value**	135 (65 – 288)
**Troponin on Admission**	3.65 (0.415 – 15.25)
**S. Creatinine on Admission**	0.97 (± 0.46) 0.9 (0.7 – 1.1)
**Creatinine Clearance**	95.69 (± 33.67) 96.5 (73 – 121)

Continuous data are presented as mean (±SD) and median (IQR).

Categorical data are presented as count (%).

Regarding the type of myocardial infarction, 117 (78.5%) had acute STEMI, while 32 (21.5%) had NSTEMI. As per Killip Classification, 125 (83.9%) were Killip Class I, 15 (10.1%) were Killip Class II, 2 (1.3%) were Killip Class III, and 7 (4.7%) were Killip Class IV. The mean ± SD in-hospital GRACE score was 149.62 ± 29.89, and the mean ± SD post-discharge GRACE score was 116.78 (± 25.15).

### Angiographic, procedural, and clinical findings

Table 2 shows the angiographic and procedural data within the study population. Among our sample, 24 (16.1%) were completely revascularized, while 125 (83.9%) had residual lesions within the study period.

**Table 2:** Angiographic data of the study sample

Angiographic Data	
**Dominance**	**Right:** 136 (91.3%) **Left:** 13 (8.7%)
**Number of Initial Lesions**	3.09 (± 1.31)
**Number of Residual Lesions**	1.87 (± 1.51)
**Culprit Vessel**	**RCA:** 52 (34.9%) **LMCA:** 2 (1.3%) **LAD:** 72 (48.3%) **LCx:** 23 (15.4%)
**Baseline Culprit TIMI Flow**	**0-1:** 95 (63.7%) **2-3:** 54 (36.3%)
**Post-procedural Culprit TIMI Flow**	**0-1:** 4 (2.7%) **2-3:** 145 (97.3%)
**Cases with LMCA Lesions**	9 (6%)
**Proximal RCA**	55 (36.9%)
**Proximal LAD**	69 (46.3%)
**Proximal LCx**	57 (38.3%)
**Staged PCI**	30 (20.1%)
**Interval Between PCI Sessions in Months**	4.98 (± 3.43) 4 (3 – 6.25)
**bSS**	23.61 (± 10.54) 23 (16.75 – 27.5)
**rSS**	10.41 (± 11) 8 (2 – 13.5)
**SRI %**	60.81% (± 29.49) 65.22% (38.32 – 87.69)
**Complete Revascularization**	24 (16.1%)
**Number of Stents Used**	1.85 (± 0.96)

Continuous data are presented as mean (±SD) and median (IQR).

Categorical data are presented as count (%).

Table 3 shows the in-hospital and follow-up events. Of all the patients included, 22 (14.8%) had an in-hospital MACE, and 39 (34.2%) of the patients who met the follow-up cumulative end-points had a recorded MACE during the follow-up period, starting from the index procedure. Regarding cardiac mortality, 23 (15.4%) of the patients died during the study period, including 7 (4.7%) in-hospital deaths and 16 (10.7%) deaths that occurred later in the follow-up period, all assumed to be cardiac.

**Table 3:** In-hospital and follow-up outcomes of the overall study sample

In-Hospital Events	
**In-Hospital Composite MACE**	22 (14.8%)
**In-Hospital Heart Failure**	13 (8.7%)
**In-Hospital Non-fatal MI**	1 (0.7%)
**In-Hospital Target Lesion Revascularization**	1 (0.7%)
**In-Hospital Death**	7 (4.7%)
**Non-MACE In-Hospital Adverse Events**	
**In-Hospital Bleeding**	1 (0.7%)
**Acute Kidney Injury**	11 (7.4%)
**Follow-up Events (*n* = 114)**	
**Follow-Up Cumulative MACE**	39 (34.2%)
**Cumulative MACE End-points**	Death: 23 (22.1%) TLR: 10 (8.8%) Non-Fatal MI: 8 (7%) Heart Failure: 13 (11.4%)

Continuous data are presented as mean (±SD) and median (IQR)

Categorical data are presented as count (%)

The study patients were divided by rSS and SRI into three groups, based on rSS and SRI tertiles, as illustrated in Tables 4 and 5, respectively. Patient characteristics were compared across the three groups of both parameters. Among the different clinical characteristics, patients were significantly older among the higher rSS tertiles. STEMI patients were more frequent among the highest tertile, while NSTEMI patients were more among the lowest tertile. There was no statistically significant difference in symptom onset to presentation time values between rSS as well as SRI tertiles. In-hospital and post-discharge GRACE scores significantly differed across the tertiles, with the highest values among the 3rd tertile. In-hospital mortality was highest among the third tertile (*p* = 0.03, *p* = 0.02, respectively). Moreover, at the 2-year follow-up, cumulative MACE was highest among the third tertile (*p* < 0.001), mostly from cardiac death.

**Table 4:** Characteristics of the study sample, categorized by rSS tertiles

**Variable**	**1st rSS Tertile (≤4)** **(*n*** **= 51)**	**2nd rSS Tertile (4 – 12)** **(*n*** **= 55)**	**3rd rSS Tertile (> 12)** **(*n*** **= 43)**	***P*** **value**
**Age**^a^	55.41 (±11.17) 58 (47 – 64)	60.62 (±10.34) 61 (55 – 66)	65.07 (±12.25) 64 (55 – 71)	0.002*
**Sex**^b^	**Male:** 38 (74.5%)	**Male:** 36 (65.5%)	**Male:** 28 (65.1%)	0.5
**BMI**^b^	28.18 (±4.85) 27.7 (24.43 – 30.75)	28 (±3.86) 27.7 (25.7 – 29.4)	27.2 (±4.69) 26.7 (23.4 – 29.4)	0.6
**Dyslipidemia**^**$**^	25 (49%)	33 (60%)	25 (58.1%)	0.5
**Smoking**^c^	26 (51%)	25 (45.5%)	16 (37.2%)	0.5
**Hypertension**^c^	22 (43.1%)	25 (45.5%)	20 (46.5%)	0.9
**Diabetes**^c^	20 (39.2%)	20 (36.4%)	12 (27.9%)	0.5
**In-Hospital Findings**				
**Pain to Presentation Time (Hours)** ^b^	7.12 (± 5.71) 6 (3.5 – 10)	7.32 (± 6.68) 6 (2 – 10)	6.56 (± 5.77) 5 (3 – 8)	0.8
**STEMI/NSTEMI**^c^	35 (68.6%)**/**16 (31.4%)	43 (78.2%)**/**12 (21.8%)	39 (90.7%)**/**4 (9.3%)	0.034*
**KILLIP Classification**^c^	**Class I:** 45 (88.2%) **Class II:** 3 (5.9%) **Class III:** 1 (2%) **Class IV:** 2 (3.9%)	**Class I:** 42 (76.4%) **Class II:** 11 (20%) **Class III:** 1 (1.8%) **Class IV:** 1 (1.8%)	**Class I:** 38 (88.4%) **Class II:** 1 (2.3%) **Class IV:** 4 (9.3%)	0.024*
**ST Resolution**^c^ **(*n*** **= 117)**	**N/A:** 2 (5.7%) **< 30%:** 1 (2.9%) **30-70%:** 23 (65.7%) **> 70%:** 9 (25.7%) **(*n*** **= 35)**	**< 30%:** 7 (16.3%) **30-70%:** 22 (51.2%) **> 70%:** 14 (32.6%) **(*n*** **= 43)**	**N/A:** 5 (12.8%) **< 30%:** 2 (5.1%) **30-70%:** 25 (64.1%) **> 70%:** 7 (17.9%) **(*n*** **= 39)**	0.037*
**Significant ST Resolution**^c^ **(*n*** **= 117)**	39 (90.7%)	37 (84.1%)	24 (80%)	0.4
**Echocardiography**				
**Ejection Fraction %**^a^	52.47 (± 10.31) 53 (44 – 60)	52.64 (± 10.64) 54 (44 – 63)	50.13 (± 11.95) 50 (41 – 62)	0.5
**Myocardial Wall Motion Score Index**^b^	1.43 (± 0.37) 1.63 (1.13 – 1.63)	1.39 (± 0.29) 1.375 (1.12 – 1.63)	1.463 (± 0.57) 1.38 (1.25 – 1.69)	0.7
**GRACE Scores**				
**In-Hospital GRACE Score**^b^	137.45 (± 22.42) 136 (123 – 154)	151.82 (± 27.81) 148 (133 – 168)	161.79 (± 35.44) 156 (139 – 177)	0.001*
**Post-Discharge GRACE Score**^b^	105.14 (± 21.19) 106 (90.5 – 121.5)	120.15 (± 22.72) 119 (103 - 131)	126.67 (± 27.78) 123 (105 – 151)	< 0.001*
**Laboratory Data**				
**Total CK on Admission**^b^	1395 (± 1930.71) 559 (148 – 1866)	1344.89 (± 1673.4) 548 (203 – 1717)	1419.44 (± 1887.02) 964 (359.25 – 1816.5)	0.4
**Peak CK value**^b^	1768.09 (± 2070.53) 879 (258 – 2446)	1797.66 (± 2066.65) 1079 (330 – 1986)	1835.5 (± 1898.92) 1252.5 (619 – 2783)	0.4
**CK-MB on Admission**^b^	168.75 (± 203.47) 80.50 (36.25 – 241.75)	143.18 (± 150.74) 71.5 (38 – 199.75)	256.46 (± 411.99) 135 (46 – 269)	0.2
**Peak CK-MB value**^b^	195.58 (± 204.91) 116.50 (53.25 – 286.25)	182.18 (± 186.42) 112 (52 – 247.75)	301.89 (± 407.24) 193 (93 – 325)	0.4
**Troponin**^b^	7.55 (± 12.77) 1.59 (0.35 – 10.8)	14.57 (± 31.04) 2.9 (0.66 – 12.83)	22.001 (± 25.2) 7.11 (2.35 – 50)	0.2
**S. Urea on Admission**^b^	5.8 (± 2.9) 5 (4 – 7)	6.289 (± 2.75) 6 (4.5 – 7)	6.3 (± 3.1) 6 (5 – 7)	0.3
**S. Creatinine on Admission**^b^	0.88 (± 0.26) 0.9 (0.7 – 1)	0.9369 (±0.28) 0.9 (0.8 – 1.1)	1.119 (± 0.76) 1 (0.7 – 1.3)	0.1
**Creatinine Clearance**^a^	105.67 (± 28.43) 100 (84 – 128)	100.24 (± 31.23) 104 (75.5 – 122.75)	81.03 (± 36.7) 84 (47 – 110.5)	0.018*
**Angiographic Data**				
**Dominance**^c^	**Right:** 47 (92.2%) **Left:** 4 (7.8%)	**Right:** 48 (87.3%) **Left:** 7 (12.7%)	**Right:** 41 (95.3%) **Left:** 2 (4.7%)	0.4
**Number of Initial Lesions**^b^	2.27 (± 0.49)	3.22 (± 1.12)	3.91 (± 1.62)	< 0.001*
**Number of Residual Lesions**^b^	0.55 (± 0.54)	2.05 (± 0.91)	3.19 (± 1.62)	< 0.001*
**Culprit Vessel**^c^	**RCA:** 10 (19.6%) **LMCA:** 1 (2%) **LAD:** 32 (62.7%) **LCx:** 8 (15.7%)	**RCA:** 18 (32.7%) **LM:** 0 (0%) **LAD:** 28 (50.9%) **LCx:** 9 (16.4%)	**RCA:** 24 (55.8%) **LM:** 1 (2.3%) **LAD:** 12 (27.9%) **LCx:** 6 (14%)	0.009*
**Baseline Culprit TIMI Flow**^c^	0-1: 26 (51%) 2-3: 25 (49%)	0-1: 37 (67.3%) 2-3: 18 (32.7%)	0-1: 32 (74.4%) 2-3: 11 (25.6%)	0.049*
**Post-procedural Culprit TIMI Flow**^c^	0-1: 0 (0%) 2-3: 51 (100%)	0-1: 0 (0%) 2-3: 55 (100%)	0-1: 4 (9.3%) 2-3: 39 (90.7%)	0.007*
**Cases with LMCA Lesions**^c^	1 (2%)	0 (0%)	8 (18.6%)	< 0.001*
**Proximal RCA**^c^	13 (25.5%)	19 (34.5%)	23 (53.5%)	0.018*
**Proximal LAD**^c^	22 (43.1%)	21 (38.2%)	26 (60.5%)	0.08
**Proximal LCx**^c^	11 (21.6%)	19 (34.5%)	27 (62.8%)	< 0.001*
**Staged PCI**^c^	15 (29.4%)	12 (21.8%)	3 (7%)	0.024*
**Interval Between PCI Sessions in Months**^b^	4.3 (± 3.3) 3 (3 – 5)	6.25 (± 3.72) 6 (3 – 10)	3.33 (± 0.58) 3 (N/A)	0.3
**bSS**^b^	17.333 (± 6.07) 18 (12 – 22)	22.191 (± 7.19) 22 (16 – 27)	32.884 (± 11.96) 29.5 (24.5 – 39)	< 0.001*
**SRI %**^b^	91.34% (± 10.67) 94.44% (84.62 – 100)	57.429% (± 18.96) 59.09% (50 – 71.43)	28.93% (± 16.7) 29.73 (16.87 – 43.48)	< 0.001*
**Number of Stents Used**^b^	2.02 (± 0.91)	2.02 (± 1.03)	1.42 (± 0.82)	0.021*
**In-Hospital Events**				
**In-Hospital Composite MACE**^c^	4 (7.8%)	10 (18.2%)	8 (18.6%)	0.2
**In-Hospital Heart Failure**^c^	1 (2%)	10 (18.2%)	2 (4.7%)	0.005*
**In-Hospital Re-Infarction**^c^	0 (0%)	0 (0%)	1 (2.3%)	0.3
**In-Hospital TLR**^c^	0 (0%)	0 (0%)	1 (2.3%)	0.3
**In-Hospital Death**^c^	2 (3.9%)	0 (0%)	5 (11.6%)	0.024*
**Non-MACE In-Hospital Adverse Events**				
**In-Hospital Bleeding**^c^	1 (2%)	0 (0%)	0 (0%)	0.6
**Acute Kidney Injury**^c^	3 (6.1%)	5 (9.1%)	3 (7.7%)	0.9
**Follow-Up Events**				
**Variable**	**1st rSS Tertile (≤4)** **(*n*** **= 35)**	**2nd rSS Tertile (4 – 12)** **(*n*** **= 42)**	**3rd rSS Tertile (> 12)** **(*n*** **= 37)**	***P*** **value**
**Follow-Up Cumulative MACE**^c^	7 (20%)	14 (33.3%)	18 (48.6%)	0.037*
**Overall Cumulative Adverse Events**				
**Death**^c^	4 (12.1%)	3 (8.8%)	16 (43.2%)	< 0.001*
**Heart Failure**^c^	1 (2.9%)	10 (23.8%)	2 (5.4%)	0.006*
**Non-Fatal MI**^c^	1 (2.9%)	1 (2.4%)	6 (16.2%)	0.029*
**TLR**^c^	4 (11.4%)	4 (9.5%)	2 (5.4%)	0.7

Continuous data are presented as mean (±SD) and median (IQR)

Categorical data are presented as count (%)

^a^ Parametric continuous data distributions are compared using one-way ANOVA

^b^ Non-parametric continuous data distributions are compared using the Kruskal-Wallis H test

^c^ Categorical data distributions are compared using Pearson’s Chi-square test, and the Monte-Carlo method is used for data that failed to meet the test assumptions

* Statistically significant difference

**Table 5:** Characteristics of the study sample, categorized by SRI tertiles

**Variable**	**1st SRI % Tertile** **(≤ 47.06)** **(*n*** **= 48)**	**2nd SRI % Tertile** **(47.06–78.18)** **(*n*** **= 52)**	**3rd SRI % Tertile** **(> 78.18)** **(*n*** **= 49)**	***P*** **value**
**Age**^a^	64.31 (±12.18) 63.5 (55.25 – 71)	59.08 (±10.24) 60.5 (54.25 – 65)	57.12 (±11.93) 58 (49.5 – 64)	0.007*
**Sex**^a^	**Male:** 31 (64.6%)	**Male:** 36 (69.2%)	**Male:** 35 (71.4%)	0.8
**BMI**^a^	27.48 (±4.84) 26.9 (24.3 – 29.4)	27.903 (±3.87) 27.7 (24.95 – 29.4)	28.05 (±4.7) 27.7 (24.5 – 29.7)	0.9
**Dyslipidemia**^b^	27 (56.3%)	33 (63.5%)	23 (46.9%)	0.2
**Smoking**^b^	18 (37.5%)	25 (48.1%)	24 (49%)	0.6
**Hypertension**^b^	22 (45.8%)	23 (44.2%)	22 (44.9%)	0.99
**Diabetes**^b^	15 (31.3%)	20 (38.5%)	17 (34.7%)	0.8
**In-Hospital Findings**				
**Pain to Presentation Time (Hours)**^a^	7.29 (± 6.11) 6 (4 – 7.75)	5.89 (± 4.13) 5.5 (2 – 9.75)	7.86 (± 7.45) 5 (3 – 12)	0.6
**STEMI/NSTEMI**^b^	38 (79.2%)/10 (21.8%)	45 (86.5%)/7 (13.5%)	34 (69.4%)/15 (30.6%)	0.1
**ST Resolution**^b^ **(*n*** **= 117)**	N/A: 4 (10.5%) < 30%: 4 (10.5%) 30 – 70%: 25 (65.8%) > 70%: 5 (13.2%) **(*n*** **= 38)**	N/A: 1 (2.2%) < 30%: 4 (8.9%) 30 – 70%: 25 (55.6%) > 70%: 15 (33.3%) **(*n*** **= 45)**	N/A: 2 (5.9%) < 30%: 2 (5.9%) 30 – 70%: 20 (58.8%) > 70%: 10 (29.4%) **(*n*** **= 34)**	0.4
**Significant ST Resolution**^b^ **(*n*** **= 117)**	30 (78.9%)	40 (88.9%)	30 (88.2%)	0.4
**KILLIP Classification**^b^	Class I: 43 (89.6%) Class II: 1 (2.1%) Class III: 0 (0%) Class IV: 4 (8.3%)	Class I: 41 (78.8%) Class II: 10 (19.2%) Class III: 0 (0%) Class IV: 1 (1.9%)	Class I: 41 (83.7%) Class II: 4 (8.2%) Class III: 2 (4.1%) Class IV: 2 (4.1%)	0.017*
**Echocardiography**				
**Ejection Fraction %**^a^	53.23 (± 11.98) 52.5 (47 – 63)	51.81 (± 11.25) 53.5 (41.5 – 60)	50.74 (± 9.38) 51 (43 – 59)	0.6
**Myocardial Wall Motion Score Index**^a^	1.379 (± 0.36) 1.25 (1.13 – 1.5)	1.436 (± 0.29) 1.375 (1.25 – 1.75)	1.448 (± 0.37) 1.375 (1.19 – 1.75)	0.3
**GRACE Scores**				
**In-Hospital GRACE Score**^a^	157.41 (± 35.21) 150 (136.75 – 175.25)	149.12 (± 25.48) 148.5 (129 – 167.5)	142.87 (± 27.85) 139 (123 – 156)	0.07
**Post-Discharge GRACE Score**^a^	123.82 (± 27.1) 120 (105 – 141)	116.75 (± 20.03) 118 (102.25 – 129)	110.23 (± 27.02) 106 (91 – 124)	0.03*
**Laboratory Data**				
**Total CK on Admission**^a^	1211.64 (± 1823.93) 611 (217 – 1488)	1436.94 (± 1673.42) 664.5 (298 – 2236.75)	1485.41 (± 1986.43) 548 (172.5 – 2168)	0.7
**Peak CK value**^a^	1542.54 (± 1852.77) 1145 (552 – 1930)	1923.75 (± 2042.19) 1189 (363.75 – 2828)	1893.49 (± 2128.73) 1220 (287 – 2560)	0.9
**CK-MB on Admission**^a^	188.02 (± 287.06) 94 (40.5 – 200)	192.34 (± 296.36) 115 (38.5 – 281)	168.68 (± 207.09) 79 (37.25 – 241.75)	0.9
**Peak CK-MB value**^a^	223.41 (± 289.12) 128 (71.5 – 270.5)	239.88 (± 311.63) 150.5 (62.75 – 334)	193.48 (± 206.95) 116.5 (52.5 – 277.5)	0.7
**Troponin**^a^	19.861 (± 36.72) 4.65 (0.77 – 23.75)	11.696 (± 17.46) 3.8 (0.36 – 16.5)	9.012 (± 16.07) 1.59 (0.3 – 9.6)	0.6
**S. Urea on Admission**^a^	6.1932 (± 3.11) 5 (4.13 – 7)	6.345 (± 3.02) 6 (5 – 7)	5.824 (± 2.52) 5 (4 – 7)	0.5
**S. Creatinine on Admission**^a^	1.101 (± 0.73) 0.91 (0.7 – 1.3)	0.924 (± 0.27) 0.9 (0.8 – 1.09)	0.893 (± 0.25) 0.9 (0.7 – 1.1)	0.3
**Creatinine Clearance**^a^	82.38 (± 34.55) 88.5 (51.75 – 107)	101.44 (± 33.36) 101 (80 – 129.75)	103.72 (± 29.45) 101.5 (85.5 – 118.75)	0.016*
**Angiographic Data**				
**Dominance**^b^	Right: 46 (95.8%) Left: 2 (4.2%)	Right: 44 (84.6%) Left: 8 (15.4%)	Right: 46 (93.9%) Left: 3 (6.1%)	0.1
**Number of Initial Lesions**^a^	3.88 (± 1.58) 3 (3 – 4.75)	3.04 (± 1.08) 3 (2 – 4)	2.39 (± 0.67) 2 (2 – 3)	< 0.001*
**Number of Residual Lesions**^a^	3.13 (± 1.59)	1.96 (± 0.86)	0.53 (± 0.54)	< 0.001*
**Culprit Vessel**^b^	RCA: 30 (62.5%) LM: 1 (2.1%) LAD: 11 (22.9%) LCx: 6 (12.5%)	RCA: 14 (26.9%) LM: 0 (0%) LAD: 27 (51.9%) LCx: 11 (21.2%)	RCA: 8 (16.3%) LM: 1 (2%) LAD: 34 (69.4%) LCx: 6 (12.2%)	< 0.001*
**Baseline Culprit TIMI Flow**^b^	0-1: 29 (60.4%) 2-3: 19 (39.6%)	0-1: 37 (71.2%) 2-3: 15 (28.8%)	0-1: 29 (59.2%) 2-3: 20 (40.8%)	0.4
**Post-procedural Culprit TIMI Flow**^b^	0-1: 4 (8.3%) 2-3: 44 (91.7%)	2-3: 52 (100%)	2-3: 49 (100%)	0.01*
**Cases with LMCA Lesions**^b^	7 (14.6%)	1 (1.9%)	1 (2%)	0.009*
**Proximal RCA**^b^	25 (52.1%)	18 (34.6%)	12 (24.5%)	0.017*
**Proximal LAD**^b^	21 (43.8%)	23 (44.2%)	25 (51%)	0.7
**Proximal LCx**^b^	24 (50%)	21 (40.4%)	12 (24.5%)	0.033*
**Staged PCI**^b^	2 (4.2%)	10 (19.2%)	18 (36.7%)	< 0.001*
**Interval Between PCI Sessions in Months**^a^	3.5 (± 0.71)	6.9 (± 3.73)	4.083 (± 3.06)	0.1
**bSS**^a^	28 (± 13.28) 25.25 (18.63 – 31)	23.183 (± 9.47) 24 (15.5 – 29)	19.776 (± 6.35) 19 (15.5 – 23.75)	0.003*
**rSS**^a^	21.219 (± 12.2) 17 (12.13 – 25.75)	8.808 (± 4.66) 8.5 (5.25 – 11.75)	1.52 (± 1.93) 1 (0 – 3)	< 0.001*
**Number of Stents Used**^a^	1.44 (± 0.74)	1.92 (± 1.1)	2.16 (± 0.87)	0.002*
**In-Hospital Events**				
**In-Hospital Composite MACE**^b^	5 (10.4%)	8 (15.4%)	9 (18.4%)	0.5
**In-Hospital Heart Failure**^b^	2 (4.2%)	4 (7.7%)	7 (14.3%)	0.2
**In-Hospital Non-fatal MI**^b^	1 (2.1%)	0 (0%)	0 (0%)	0.3
**In-Hospital TLR**^b^	1 (2.1%)	0 (0%)	0 (0%)	0.3
**In-Hospital Death**^b^	4 (8.3%)	1 (1.9%)	2 (4.1%)	0.2
**Non-MACE In-Hospital Adverse Events**				
**In-Hospital Bleeding**^b^	0 (0%)	0 (0%)	1 (2%)	0.7
**Acute Kidney Injury**^b^	3 (6.3%)	2 (3.8%)	6 (12.2%)	0.3
**Follow-Up Data**				
**Variable**	**1st SRI Tertile** **(≤ 47.06)** **(*n*** **= 40)**	**2nd SRI Tertile** **(47.06–78.18)** **(*n*** **= 35)**	**3rd SRI Tertile** **(> 78.18)** **(*n*** **= 39)**	***P*** **value**
**Adverse Events**				
**Follow-Up Cumulative MACE**^b^	17 (42.5%)	10 (28.6%)	12 (30.8%)	0.4
**Overall Cumulative Adverse Events**				
**Death**^b^	13 (32.5%)	5 (14.3%)	5 (12.8%)	0.054
**Non-fatal MI**^b^	5 (12.5%)	2 (5.7%)	1 (2.6%)	0.2
**TLR**^b^	2 (5%)	3 (8.6%)	5 (12.8%)	0.4
**Heart Failure**^b^	3 (7.5%)	6 (17.1%)	4 (10.3%)	0.4

Continuous data are presented as mean (±SD) and median (IQR).

Categorical data are presented as count (%).

^a^ Non-parametric continuous data distributions are compared using the Kruskal-Wallis H test.

^b^ Categorical data distributions are compared using Pearson’s Chi-square test, and the Monte-Carlo method is used for data that failed to meet the test assumptions.

* Statistically significant difference.

As shown in Table 5, patients were categorized according to SRI tertiles into 3 groups. Those in the first tertile were significantly older, and had worse Killip class and higher GRACE score. Regarding clinical outcomes, although in-hospital outcomes were not significantly different, upon follow-up, mortality was significantly higher among first SRI tertile.

### Analysis of outcomes

As shown in Table 6, The univariable logistic regression analysis showed that the female sex, hypertension, acute kidney injury (AKI), lower LV Ejection Fraction, BSS, and rSS were associated with In-Hospital composite MACE. Multivariable analysis showed that female sex, hypertension, and lower LV Ejection Fraction were independent predictors for In-Hospital composite MACE. Further analysis of rSS and SRI found that both were not statistically significant predictors of in-hospital mortality (OR for rSS = 0.95, 95% CI 0.86 – 1.06, *p* = 0.346, OR for SRI = 1.01, 95% CI 0.98 – 1.04, *p* = 0.527).

**Table 6:** Univariate and multivariate logistic regression models for predictors of in-hospital composite MACE

Variable	Univariable Odds ratio	95% C.I.		*P* value	Multivariable Odds ratio	95% C.I.		*P* value
		lower	upper			lower	upper	
**Age**	1.03	0.99	1.08	0.1	0.95	0.89	1.02	0.1
**Sex (Female)**	3.15	1.25	7.96	0.015*	11.64	1.85	73.41	0.009*
**Hypertension**	2.44	0.96	6.24	0.06	10.21	1.65	63.33	0.013*
**Diabetes**	0.66	0.24	1.81	0.4	0.39	0.07	2.32	0.3
**LVEF**	0.86	0.79	0.92	< 0.001*	0.79	0.70	0.89	< 0.001*
**AKI**	5.71	1.46	22.36	0.012*	3.98	0.74	21.46	0.1
**BSS**	1.06	1.02	1.10	0.005*	1.07	0.95	1.21	0.2
**rSS**	1.04	1.00	1.08	0.032*	0.98	0.89	1.08	0.7
**SRI**	0.99	0.98	1.01	0.3	–	–	–	–

C-index = 0.94 (0.90 – 0.98)

*LVEF* Left ventricular ejection fraction, *AKI* Acute Kidney Injury, *BSS* Baseline SYNTAX Score, *rSS* Residual SYNTAX Score

**Fig. 2:**
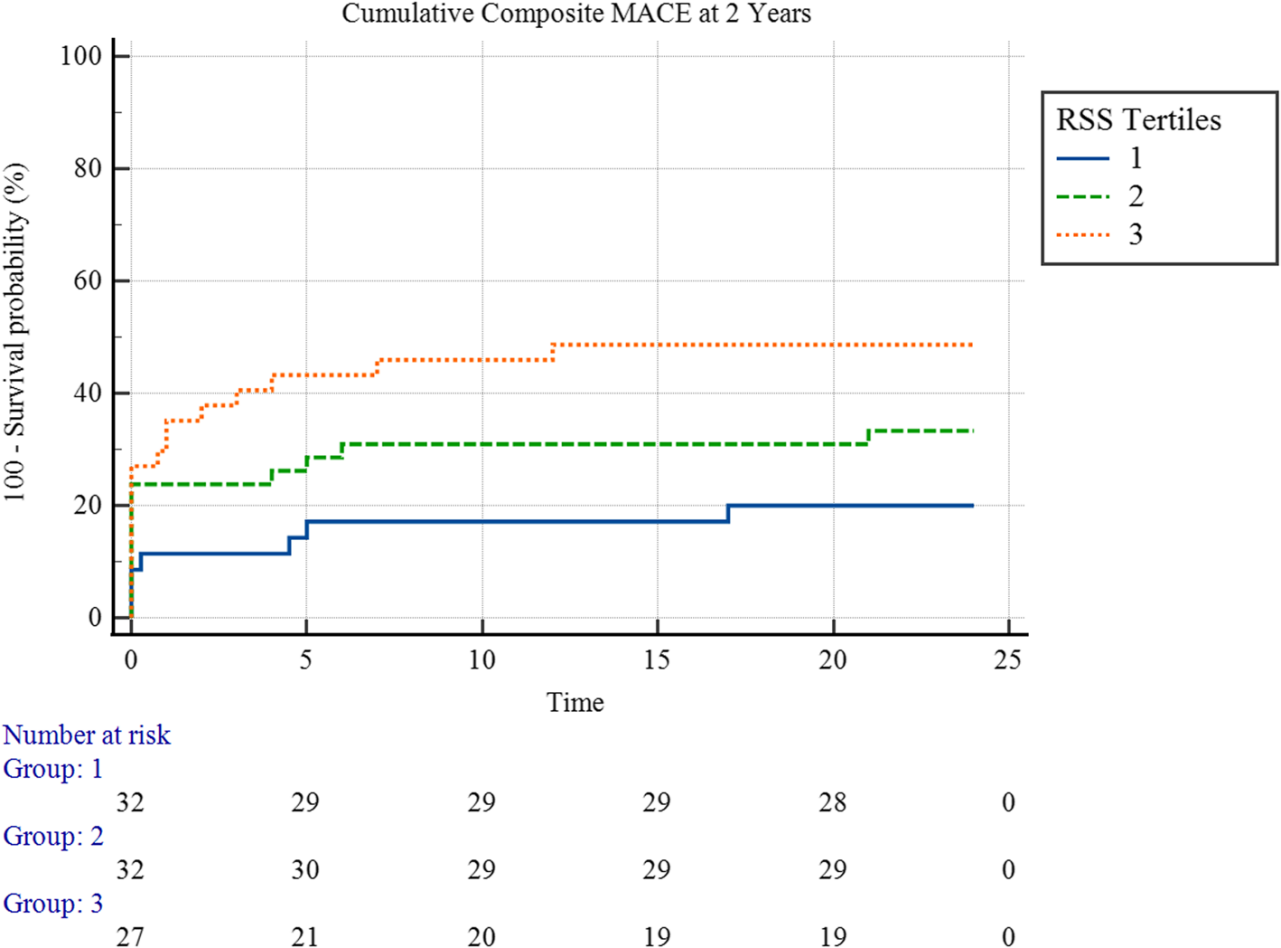
Kaplan-Meyer hazard curves for 2-year Cumulative MACE hazard, stratified by rSS Tertiles

Figure 2 showed that there was an overall statistically significant difference in the 2-year cumulative MACE hazard between the three rSS tertiles (χ^2^ = 6.84, *p* = 0.03), with a statistically significant pairwise difference between the first and the third tertiles (χ^2^ = 6.76, *p* = 0.009) and, while the pairwise difference between the first and the second tertiles was not statistically significant (χ^2^ = 1.75, *p* = 0.2), nor between the second and the third tertiles (χ^2^ = 1.92, *p* = 0.2).

The mean survival time for cumulative composite MACE at 2 years for the first tertile was 13.97 (95% CI 17.16 – 22.77) months, and for the second tertile, it was 16.86 (95% CI 13.65 – 20.06) months, and 13.13 (95% CI 9.50 – 16.81) for the third tertile.

**Fig. 3:**
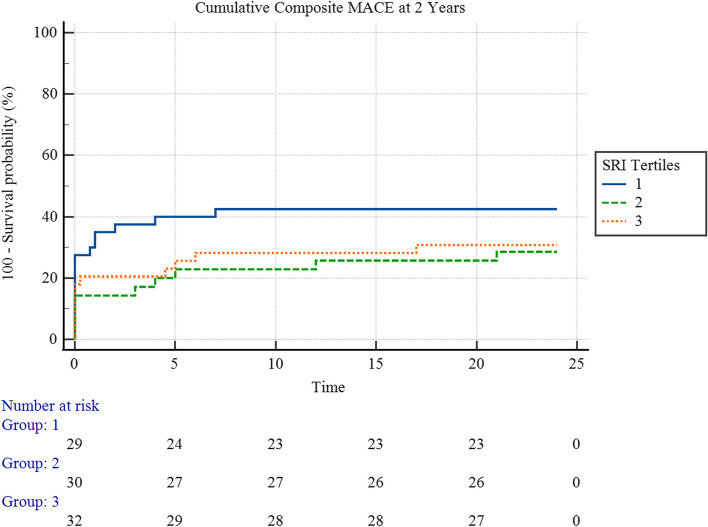
Kaplan-Meyer hazard curves for 2-year cumulative MACE hazard, stratified by SRI Tertiles

Figure 3 showed neither an overall statistically significant difference in the 2-year cumulative MACE hazard between the three SRI tertiles (χ^2^ = 2.28, *p* = 0.3) nor a statistically significant pairwise difference between any pair of the tertiles, as illustrated.

The mean survival time for cumulative composite MACE at 2 years for the first tertile was 14.19 (95% CI 10.64 – 17.75) months, and for the second tertile, it was 18.43 (95% CI 15.29 – 21.57) months, and 17.46 (95% CI 14.26 – 18.56) for the third tertile.

Univariable Cox regression analysis for the angiographic parameters (Table 7) showed that higher rSS was a significant predictor of 2-year cumulative MACE, unlike the SRI, which was not statistically significant. In addition, multivariable Cox regression analysis for different clinical and angiographic parameters (Tables 8 and 9) showed that female sex, hypertension, and lower EF were predictors of cumulative MACE in 2 years, along with rSS. Still, when rSS was substituted with SRI, it was found to be a nonsignificant predictor with a very close *p*-value to significance (*p* = 0.003 for rSS, *p* = 0.09 for SRI). Since rSS is a component of SRI, it was better to evaluate each in a separate model to avoid collinearity and ensure the accuracy of prediction models.

**Table 7:** Cox Regression Models for Cumulative MACE at 2 Years

Variable	HR (95% CI)	*P* value	C-index (95% CI)
**Angiographic Scores (Univariable)**			
**rSS**	1.04 (1.02 – 1.06)	< 0.001*	0.64 (0.55 - 0.73)
**SRI**	0.99 (0.98 – 1.00)	0.06	0.60 (0.51 – 0.698)

**Table 8:** Multivariate Cox Regression Model for 2-Year cumulative MACE (Including Patient Risk Factors and rSS)

Variable	HR (95% CI)	*P* value
**Age**	1.02 (0.98 - 1.06)	0.4
**Female Sex**	2.36 (1.10- 5.05)	0.028*
**BMI**	1.01 (0.92 - 1.09)	0.9
**Hypertension**	2.14 (1.06 - 4.35)	0.035*
**Diabetes**	0.82 (0.38 - 1.77)	0.6
**LV Ejection Fraction**	0.91 (0.86 - 0.96)	< 0.001*
**Myocardial WMSI**	0.46 (0.10 - 2.12)	0.3
**Creatinine Clearance**	1.01 (0.99 - 1.03)	0.3
**rSS**	1.04 (1.01 - 1.06)	0.003*

C-index = 0.802 (0.73 - 0.87)

**Table 9:** Multivariate Cox Regression Model for 2-Year cumulative MACE (Including Patient Risk Factors and SRI)

Variable	HR (95% CI)	*P* value
**Age**	1.02 (0.98 - 1.06)	0.4
**Female Sex**	2.13 (1.02 - 4.47)	0.04*
**BMI**	0.99 (0.91 - 1.08)	0.9
**Hypertension**	2.08 (1.05 - 4.14)	0.04*
**Diabetes**	0.80 (0.37 - 1.70)	0.6
**LV Ejection Fraction**	0.91 (0.87 - 0.96)	0.001*
**Myocardial WMSI**	0.51 (0.11 - 2.32)	0.4
**Creatinine Clearance**	1.01 (0.99 - 1.02)	0.3
**SRI**	0.99 (0.98 to 1.00)	0.09

C-index = 0.79 (0.73 - 0.86)

**Fig. 4:**
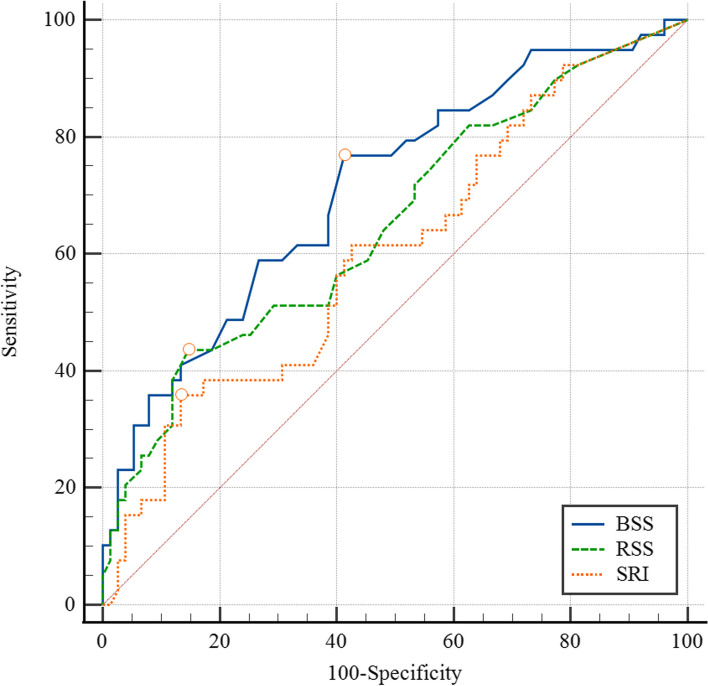
shows the comparative performance of different angiographic scores in predicting 2-year cumulative MACE

As shown in Fig. 4, rSS was non-inferior to bSS in the predictive performance of cumulative MACE among the angiographic scores (AUC = 0.65, *p* = 0.006) at a cut-off value of 14 with 43.6% sensitivity and 85.3% specificity. Meanwhile, the SRI demonstrated inadequate discrimination ability to predict the incidence of cumulative MACE (AUC = 0.604, *p* = 0.07).

### Analysis of STEMI patients only

**Fig. 5:**
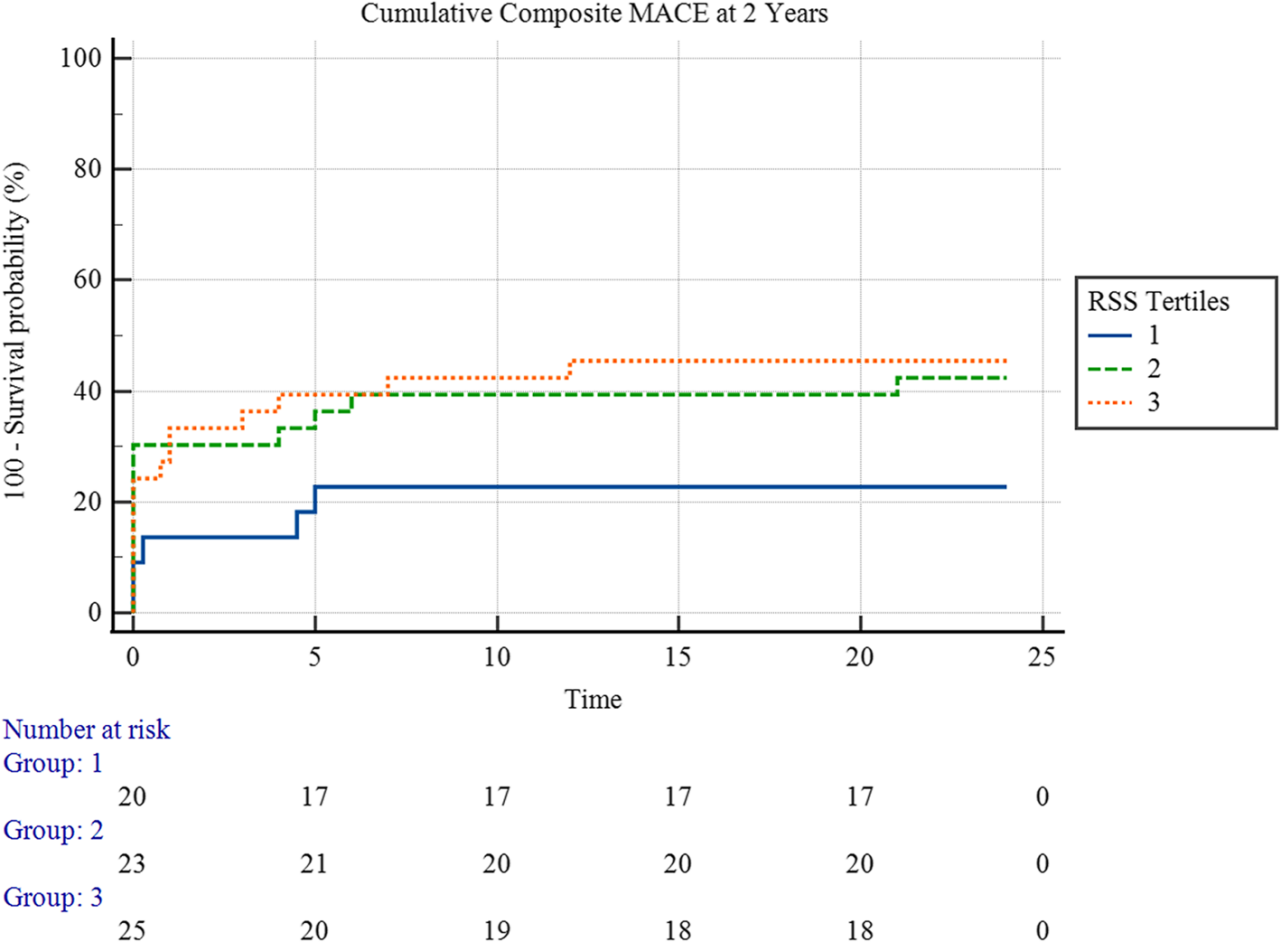
Kaplan-Meyer hazard curves for 2-year Cumulative MACE hazard in STEMI patients only, stratified by rSS Tertiles

**Fig. 6:**
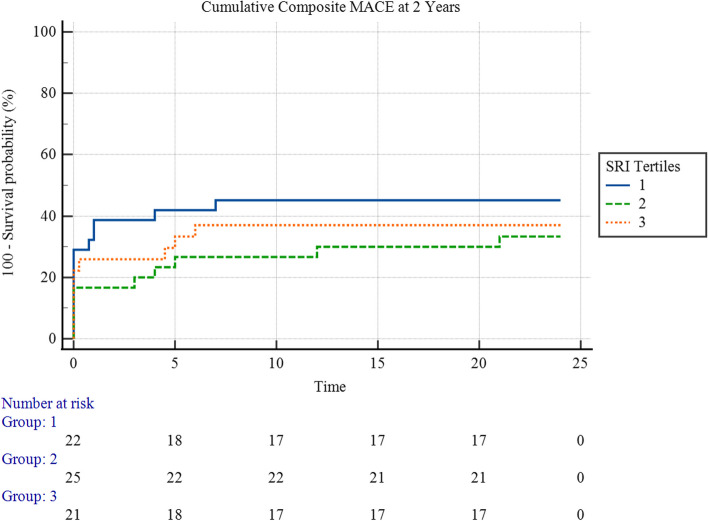
Kaplan-Meyer hazard curves for 2-year cumulative MACE hazard in STEMI patients only, stratified by SRI Tertiles

Since most of the study patients presented with STEMI, we conducted a separate analysis for this group. We found consistently that neither rSS nor SRI were significant predictors of in-hospital MACE (rSS: OR = 1.02, 95% CI 0.98 – 1.06, *p* = 0.3, SRI: OR = 0.99, 95% CI 0.98 – 1.01, *p* = 0.4). Further study of this subgroup’s long-term outcomes was conducted via survival and Cox regression analyses (Figs. 5, 6 and Tables 10, 11 and 12).

**Table 10:** Cox Regression Models for cumulative MACE at 2 Years in STEMI patients only

Variable	HR (95% CI)	*P* value	C-index (95% CI)
**Angiographic Scores (Univariable)**			
**rSS**	1.03 (1.00 – 1.06)	0.032*	0.61 (0.52 – 0.71)
**SRI**	0.99 (0.98 – 1.00)	0.2	0.57 (0.48 – 0.68)

**Table 11:** Multivariate Cox Regression Model for 2-Year cumulative MACE in STEMI patients only (Including Patient Risk Factors and rSS)

Variable	HR (95% CI)	*P* value
**Age**	1.03 (0.99 – 1.08)	0.1
**Female Sex**	2.82 (1.21 – 6.59)	0.02*
**BMI**	1.03 (0.94 – 1.13)	0.6
**Hypertension**	2.09 (1.01 – 4.35)	0.048*
**Diabetes**	0.65 (0.27 – 1.58)	0.3
**LV Ejection Fraction**	0.92 (0.87 – 0.98)	0.007*
**Myocardial WMSI**	0.82 (0.12 – 5.63)	0.8
**Creatinine Clearance**	1.02 (1.00 – 1.04)	0.03*
**rSS**	1.03 (1.00 – 1.06)	0.03*

C-index = 0.797 (0.73 – 0.89)

**Table 12:** Multivariate Cox Regression Model for 2-Year cumulative MACE in STEMI patients only (Including Patient Risk Factors and SRI)

Variable	HR (95% CI)	*P* value
**Age**	1.03 (0.99 – 1.08)	0.1
**Female Sex**	2.44 (1.08 – 5.52)	0.03*
**BMI**	1.01 (0.92 – 1.11)	0.8
**Hypertension**	2.11 (1.03 – 4.32)	0.04*
**Diabetes**	0.62 (0.25 – 1.50)	0.3
**LV Ejection Fraction**	0.92 (0.87 – 0.98)	0.008*
**Myocardial WMSI**	0.87 (0.13 – 5.89)	0.9
**Creatinine Clearance**	1.02 (1.00 – 1.04)	0.03*
**SRI**	0.99 (0.98 – 1.00)	0.2

C-index = 0.79 (0.71 – 0.86)

Figure 5 showed no overall statistically significant difference in the 2-year cumulative MACE hazard between the three rSS tertiles (χ^2^ = 3.04, *p* = 0.2).

The mean survival time of STEMI patients for cumulative composite MACE at 2 years for the first tertile was 18.99 (95% CI 15.10 – 22.88) months, and for the second tertile, it was 14.91 (95% CI 11.09 – 18.73) months, and 13.96 (95% CI 10.13 – 17.79) months for the third tertile.

Figure 6 showed no overall statistically significant difference in the 2-year cumulative MACE hazard in STEMI patients between the three SRI tertiles (χ^2^ = 1.17, *p* = 0.6).

The mean survival time of STEMI patients for cumulative composite MACE at 2 years for the first tertile was 13.61 (95% CI 9.55 – 17.66) months, and for the second tertile, it was 17.5 (95% CI 13.95 – 21.06) months, and 15.70 (95% CI 11.58 – 19.82) months for the third tertile.

Univariable Cox regression analysis for the angiographic parameters in STEMI patients (Table 10) showed that higher rSS was a significant predictor of 2-year cumulative MACE, unlike the SRI, which was not statistically significant. In addition, multivariable Cox regression analysis for different clinical and angiographic parameters (Tables 11 and 12) showed that female sex, hypertension, lower EF, and creatinine clearance were predictors of cumulative MACE in 2 years, along with rSS. Still, when rSS was substituted with SRI, it was found to be a nonsignificant predictor (*p* = 0.03 for rSS, *p* = 0.2 for SRI). Since rSS is a component of SRI, it was better to evaluate each in a separate model to avoid collinearity and ensure the accuracy of prediction models.

## Discussion

In a cohort of patients with STEMI and NSTEMI with muli-vessel affection, we found that neither the rSS nor the SRI were significant predictors of in-hospital MACE or all-cause mortality. However, it was found that the rSS was found to be a highly significant predictor of two-year follow-up MACE. This was consistent in a subgroup analysis of STEMI-only patients.

The SYNTAX score has been developed as an approach to quantify coronary artery disease’s burden in a numeric way that could be effectively utilized as an independent predictor of mortality and major adverse cardiovascular events [24]. While incomplete revascularization negatively impacted short- and long-term outcomes, the residual burden of obstructive CAD had to be assessed to quantify the degree and complexity of obstructive lesions and subsequently conduct risk stratification of patients on this basis [25]. Hence, the residual SYNTAX score (rSS) was developed [14]. It was beneficial in risk stratification, as patients with an rSS value of over eight had a significantly higher incidence of death and adverse events within 30 days and 1 year, respectively [14].

After that, it was proposed to use both the baseline SYNTAX score (bSS) and the rSS to develop an index that can quantify the percentage of coronary obstruction treated by PCI and can be used as a tool for risk stratification and as an indicator of prognosis. As a result, the SYNTAX revascularization index (SRI) came to life. It was found that patients with an SRI of less than 70% had a higher incidence of death and MACE at 5 years, with an inverse relationship between SRI and MACE [26].

We found that the rSS and the SRI were insignificant predictors for in-hospital outcomes. Even though the rSS yielded significant outcomes in univariate logistic regression, its discrimination ability for such an application was not good enough (C-index = 0.603, *p* = 0.123). Moreover, after considering confounding factors, it was not a significant predictor of in-hospital adverse events, unlike female sex, hypertension, and left ventricular ejection fraction. These findings may go differently from similar previous studies [17, 27]. However, they concord with the study conducted by Loutfi and colleagues on a similar population in northern Egypt [28]. It is believed that the possible causes of such discrepancy could be due to differences in sample size and characteristics in different studies, along with a wide spectrum of target population involving both STEMI and NSTEMI patients. Additionally, our study had a different method of dividing patients into tertiles, which was based on the statistical tertiles and median values, unlike other studies that divided the patients into groups of fully treated patients and other groups that were divided based on a specific cut-off value, which might result in unequal distribution of patients and hence significant differences in statistical comparisons. All of such factors may result in differences in our results from other previous studies in this regard.

Upon follow-up, the rSS demonstrated a significant impact on the outcomes of patients in our study after 2 years. Even after losing a significant portion of the original study sample and resulting in a smaller sample size, it could still demonstrate significant performance in both survival analysis methods conducted in this study, with significant solid outcomes and minimal probabilities of having such occurrences due to mere chance. The Kaplan-Meyer survival analysis demonstrated that patients with rSS values over 12 had a significantly higher number of adverse events throughout the study period (Fig. 2). Moreover, Cox regression analysis proved that the rSS was a powerful predictor of MACE in univariable and multivariable settings after considering confounders (Tables 7 and 8). ROC curve analysis confirmed that the rSS is at least non-inferior to bSS in predicting cumulative MACE after 2 years (Fig. 4). Similar results were found upon analysis of data obtained from the STEMI group.

On the other hand, the SRI was less potent than the rSS in predicting MACE after 2 years. The Kaplan-Meyer survival analysis yielded insignificant outcomes for the difference between SRI tertiles, and the Cox regression analysis for cumulative MACE proved that it was neither a good predictor in a univariable manner nor good enough after taking confounders into account (Tables 7 and 9). Nevertheless, it was close to significance in this regard, with a *p*-value of 0.0846 in multivariable Cox regression analysis. A larger sample size could prove a more significant impact of SRI on patient outcomes, unlike the rSS, which performed exceptionally well in this regard even in a small sample like the one in our study.

Although it was not the main objective of our study, we noted that all patients who underwent complete revascularization (rSS = 0, SRI = 100%) did well and survived throughout the study period, with only a cumulative count of three non-fatal adverse events. The percentage of cumulative adverse events (12.5%) was close to that of the patients who underwent complete revascularization in the COMPLETE trial, which found that 13.5% of the patients with complete revascularization encountered a MACE after 3 years of follow-up. Many other studies extensively studied this aspect in more detail, and their results were similar [7–10]. Another thing to notice is that we only had a single patient with in-hospital significant bleeding, and none of the patients of the study reported follow-up significant bleeding events. The local institutional policy at the time of study conduction followed the one-year dual antiplatelet therapy (DAPT) for all PCI patients unless otherwise indicated by the treating physician based on the patient’s bleeding risk, according to the ESC current updates at this time [29]. More recent studies showed that following a short-term DAPT protocol for up to 3 months, followed by long-term P2Y12 inhibitor therapy, may result in similar treatment outcomes, reducing the patients’ bleeding risk [30, 31]. While the STOPDAPT-2 trial recommended the use of clopidogrel for long-term therapy [32], other studies indicated that patients who used ticagrelor as their P2Y12 inhibitor drug had less incidence of MACE than those who used clopidogrel [33]. In our study, the incidence of significant bleeding is considered very low (0.67%), even less than the known global incidence rates when the ACUITY major bleeding definition is used [34], mainly due to strict anti-bleeding measures, such as post-PCI vascular compression and continuous CBC monitoring, which were conducted throughout the PCI procedure and during the hospital stay in accordance to the local institutional policy. We have also noticed that there was no statistically significant difference in symptom onset to presentation time values between rSS as well as SRI tertiles.

## Limitations of the study

This is a single-center study involving a relatively small number of participants. Despite adjusting for multiple covariates, our findings might have been influenced by unmeasured confounders. In addition, a single-center study might be prone to many logistical and circumstantial obstacles, like the COVID-19 pandemic, that might hinder proper data collection and subsequent results. We highly recommend additional large-scale multicenter studies to investigate this topic in depth.

## Conclusion

Neither the residual SYNTAX score (rSS) nor the SYNTAX revascularization index (SRI) could predict in-hospital events correctly. However, the residual SYNTAX score could prove to be a significant predictor of adverse events after 2 years of follow-up, and it was found to be at least non-inferior to the baseline SYNTAX score (bSS) in the prediction of follow-up cumulative major adverse cardiovascular events. It was superior to the SRI, which could not significantly predict cumulative follow-up adverse events in a relatively small group of patients. More studies are recommended to investigate this finding further and confirm whether the SRI could prove better outcomes in larger-scale studies.

## Data Availability

All data generated or analyzed during this study are included in this published article.
